# Regional differences in the daily consumption of smoked and smokeless tobacco among adults (25-64 years) in Mozambique: 2005 versus 2014/2015

**DOI:** 10.1590/0102-311XEN029024

**Published:** 2025-02-07

**Authors:** José Ramón Enjo-Barreiro, Filipa Fontes, Sheila Tualufo, Carla Silva-Matos, Albertino Damasceno, Nuno Lunet

**Affiliations:** 1 Universidade de Santiago de Compostela, Santiago de Compostela, España.; 2 Faculdade de Medicina, Universidade do Porto, Porto, Portugal.; 3 Precancerous Lesions and Early Cancer Management Group IPO Porto Research Center, Portuguese Oncology Institute of Porto/Porto Comprehensive Cancer Centre & RISE@CI-IPOP (Health Research Network), Porto, Portugal.; 4 Departamento de Doenças Não Transmissíveis, Ministério da Saúde, Maputo, Moçambique.; 5 Unidade de Gestão do Fundo Global - Direção de Planificação e Cooperação, Ministério da Saúde, Maputo, Moçambique.; 6 Faculdade de Medicina, Universidade Eduardo Mondlane, Maputo, Moçambique.; 7 EPIUnit - Instituto de Saúde Pública, Universidade do Porto, Porto, Portugal.; 8 Laboratório para a Investigação Integrativa e Translacional em Saúde Populacional, Universidade do Porto, Porto, Portugal.

**Keywords:** Noncommunicable Diseases, Tobacco Use, Prevalence, Doenças Não Transmissíveis, Uso de Tabaco, Prevalência, Enfermedades No Transmisibles, Uso de Tabaco, Prevalencia

## Abstract

Within-country differences in the prevalence of tobacco consumption may be expected in Mozambique, as determinants of tobacco use vary considerably countrywide. We compared the daily use of smoked and smokeless tobacco in 2005 and 2014/2015 across Mozambican regions. Two surveys were conducted in Mozambique, in 2005 and 2014/2015, with representative samples of the adult population, following the World Health Organization’s STEPwise Approach to NCD Risk Factor Surveillance. Prevalence estimates were computed for daily use of different types of tobacco, stratified by regions. Data from the 2014/2015 survey were compared to those from the 2005 survey, after direct age-standardization. During the 10-year period, a significant reduction was observed in the prevalence of daily tobacco smoking among women in the Northern and men in the Southern provinces, due to the decrease in the consumption of hand-rolled cigarettes among Northern women (from 9.6% to 2.3%), and manufactured cigarettes among Southern men (from 23.7% to 11.8%). In Center and Northern regions, nonsignificant increases were observed in the consumption of manufactured cigarettes among men. The consumption of smokeless tobacco among Southern women decreased (from 3.1% to 1%). There was a decrease in the daily consumption of hand-rolled cigarettes among women in the North and of manufactured cigarettes among men in the South, as well as a potential trend towards residual smokeless tobacco consumption. However, the results suggest increases in the daily consumption of manufactured cigarettes among men in the Center and Northern regions.

## Abstract (pt)

Diferenças na prevalência do consumo de tabaco podem ser esperadas em Moçambique, considerando que os determinantes do uso de tabaco variam consideravelmente em todo o território. Este estudo comparou o uso diário de tabaco com e sem fumaça nas regiões de Moçambique. Foram realizadas duas pesquisas em Moçambique, em 2005 e 2014/2015, com amostras representativas da população adulta, seguindo a Abordagem STEPwise para a Vigilância de Fatores de Risco de Doenças Crônicas da Organização Mundial da Saúde. Estimativas de prevalência foram calculadas para o uso diário de diferentes tipos de tabaco, estratificadas por regiões de Moçambique. Os dados da pesquisa de 2014/2015 foram comparados aos da pesquisa de 2005 após padronização direta por idade. Ao longo do período de 10 anos, observou-se uma redução significativa para menos da metade na prevalência de tabagismo diário entre mulheres do Norte e homens das províncias do Sul. Isso foi devido à diminuição no consumo de cigarros enrolados entre as mulheres do Norte (de 9,6% para 2,3%) e de cigarros industrializados entre os homens do Sul (de 23,7% para 11,8%). Nas regiões Centro e Norte, observaram-se aumentos não significativos no consumo de cigarros industrializados entre os homens. Em relação ao tabaco sem fumaça, houve queda no consumo entre as mulheres do Sul (de 3,1% para 1%). Houve uma redução no consumo diário de cigarros de enrolar entre as mulheres do Norte e de cigarros industrializados entre os homens do Sul, além de uma possível tendência para o consumo residual de tabaco sem fumaça. No entanto, os resultados sugerem aumentos no consumo diário de cigarros industrializados entre homens nas regiões Centro e Norte.

## Abstract (es)

Se estiman diferencias en la prevalencia del consumo de tabaco entre la población de Mozambique, ya que los determinantes del consumo de tabaco varían considerablemente en todo el territorio. El uso diario de tabaco fumado y sin humo se comparó en distintas regiones de Mozambique entre 2005 y 2014/2015. Para ello, se realizaron dos encuestas en Mozambique, en 2005 y 2014/2015, con muestras representativas de la población adulta con base en el Enfoque STEPwise para la Vigilancia de los Factores de Riesgo de Enfermedades Crónicas de la Organización Mundial de la Salud. Las estimaciones de prevalencia se calcularon para el uso diario de diferentes tipos de tabaco, estratificadas por regiones de Mozambique. Los datos de la encuesta 2014/2015 se compararon con los de la encuesta de 2005 después de la categorización directa por edad. Durante el período de 10 años, hubo una reducción significativa de menos de la mitad en el consumo del tabaquismo diario entre las mujeres del Norte y los hombres de las provincias del Sur. Este resultado se explica por la disminución en el consumo de cigarrillos para liar entre las mujeres del Norte (del 9,6% al 2,3%) y cigarrillos industrializados entre los hombres del Sur (del 23,7% al 11,8%). En las regiones Centro y Norte, hubo aumentos no significativos en el consumo de cigarrillos industrializados entre los hombres. En relación con el tabaco sin humo, hubo una caída en el consumo entre las mujeres del Sur (del 3,1% al 1%). Hubo una reducción en el consumo diario de cigarrillos de liar entre las mujeres del Norte y cigarrillos industrializados entre los hombres del Sur, además de una posible tendencia al consumo residual de tabaco sin humo. Sin embargo, los resultados indican un aumento en el consumo diario de cigarrillos industrializados entre los hombres en las regiones Centro y Norte.

## Introduction

Smoking is the second leading risk factor for attributable deaths in both sexes, accounting for 8.7 million deaths worldwide (15.4% of all deaths in 2019) ^1^. Although the number of disability-adjusted life-years (DALYs) attributable to smoking has been decreasing since 2010, in both developed and developing countries, smoking remains the first cause of DALYs among men and the seventh among women in 2019 ^1^. The poorest and the youngest have been described as the most vulnerable to becoming smokers ^2^ ^,^ ^3^ ^,^ ^4^.

Mozambique is one of the poorest countries worldwide, with a gross domestic product of approximately USD 540 per capita in 2022 and nearly three quarters of the population living below the poverty line in 2019 ^5^. In 2022, the estimated population was 33 million, with approximately 45% aged under 15 years. The population is predominantly rural, with nearly two-thirds of rural dwellers in 2022 ^5^. Smoking was the seventh leading risk factor for attributable deaths among Mozambicans in 2019, though it does not rank among the top 10 most important causes of DALYs for both sexes ^1^.

We previously reported that at least eight out of 10 current smokers (84% women and 92% men) and current consumers of smokeless tobacco (100% of women and 80% of men) were daily consumers in 2014/2015 ^6^. Additionally, a decrease in the prevalence of daily smokers in Mozambique (from 9.1% to 3.4% and from 33.6% to 27.3% in women and men, respectively; p < 0.05); similar to daily consumption of smokeless tobacco (from 7.4% to 6%; p = 0.437 among women and from 3.5% to 1%; p < 0.05 among men), was observed between 2005 and 2014/2015 ^6^. However, within-country differences in this phenomenon may be expected, as socioeconomic determinants of tobacco consumption vary considerably across Mozambique. For instance, the proportion of illiterate individuals ranges from 6.4% in Maputo City, in the South, to 61.1% in the province of Cabo Delgado, in the North ^7^. Most of the tobacco leaf production is concentrated in the provinces of Tete and Niassa, in North Mozambique ^8^. In addition, northern provinces are predominantly matrilineal and Muslim, whereas central and southern provinces are patrilineal and most frequently Christian ^9^ ^,^ ^10^.

Therefore, in this report we broaden our previous analysis on the overall trend in tobacco use in Mozambique between 2005 and 2014/2015 to characterize the variation in daily consumption of smoked and smokeless tobacco across Mozambican regions.

## Methods

This study was based on two cross-sectional analyses of representative samples of the Mozambican population, conducted from September to November 2005, and from December 2014 to February 2015.

### Selection of participants

The methods of each study and the corresponding sampling strategies have been described in detail elsewhere ^11^ ^,^ ^12^. Briefly, both surveys were designed to obtain a representative sample of the Mozambican population, at the national and provincial levels, and according to the place of residence (urban vs. rural). Sampling procedures started with the selection of geographical clusters (95 in 2005 and 120 in 2014/2015), with probability proportional to the number of households, stratified according to province, urban/rural areas, and socioeconomic strata. One area was randomly selected within each primary sampling unit, then households (25 in 2005 and 24 in 2014/2015) were selected in each area, from updated lists of households, based on random and systematic procedures. In 2005, all subjects aged 25-64 years were invited to participate in the study. In 2014/2015, a maximum of two participants were selected from each household, one aged 15-44 years and one aged 45-64 years, whenever available; when there was more than one household member in each of these age groups, only one per group was selected, using a Kish selection grid.

In 2005, a total of 3,378 subjects aged 25-64 years were invited and 3,323 (98.4%) agreed to participate. In 2014/2015, a total of 3,277 individuals aged 15-64 years were invited and 3,119 (95.2%) agreed to participate, totaling 2,181 aged 25-64 years. Considering that only adults aged 25-64 years were evaluated in 2005, those aged less than 25 years who were evaluated in 2014/2015 were excluded from the analyses. A total of 3,110 and 2,173 participants aged 25-64 years - from the 2005 and 2014/2015 surveys, respectively - which data on tobacco use were available, were included.

### Participants’ evaluation

In both surveys, subjects were evaluated following the World Health Organization’s (WHO) STEPwise Approach to NCD Risk Factor Surveillance (STEPS) ^13^, which included a questionnaire on sociodemographic and behavioral characteristics (including smoking habits). The Portuguese version of the questionnaire was used for data collection, using standardized methods in face-to-face-interviews.

Participants were asked if they smoked any tobacco product, including cigarettes, cigars and pipe, and their frequency of consumption. In the 2005 STEPS survey, daily users of multiple types of smoked or smokeless tobacco (as applicable) were asked to report the number of units, sessions, or times, of their preferred tobacco product. In the 2014/2015 survey, daily smokers were asked the number of units or sessions (as applicable) of each type of smoked tobacco consumed per day or week, namely: manufactured cigarettes, hand-rolled cigarettes, tobacco pipes, cigars/cigarillos, shisha, and other nonspecified smoked tobacco products. Participants were also asked if they were using any smokeless tobacco products at the time of the interview, namely snuff, chewing tobacco or betel. Current consumers were asked if they used any form of smokeless tobacco on a daily basis. Daily users were asked to report the number of times, per day or week, they used each tobacco product, namely snuff, moist snuff, chewing tobacco, betel/quid, and other nonspecified tobacco product.

In 2005 only two participants reported daily pipe smoking and there were no daily users of shisha or cigars. Similarly, in 2014/2015, only two participants reported daily pipe and shisha smoking, and there was no daily consumption of cigars. Therefore, these categories were not considered in the specific analysis by type of smoked tobacco. Concerning daily use of smokeless tobacco, in 2005 only two individuals reported daily consumption of betel and one of chewing tobacco, and in 2014/2015 only one individual reported daily use of chewing tobacco and betel. Therefore, as most daily use of smokeless tobacco referred to snuff, no specific analysis by type of smokeless tobacco was carried out.

The classification of the place of residence as urban or rural and the definition of categories for complete years of education by the participants (< 1; 1-5; ≥ 6) followed the 1997 census for the 2005 survey, and the 2007 census for the 2014/2015 survey.

The 11 provinces of Mozambique were grouped in Southern (including Maputo City, which is the capital, and Maputo Province, which is adjacent to the capital), Center (Inhambane, Gaza, Sofala and Manica) and Northern (Zambézia, Tete, Nampula, Niassa, and Cabo Delgado).

### Statistical analysis

Prevalence estimates with 95% confidence intervals were computed for daily use of different types of tobacco, stratified by sex, and according to age, education, place of residence (urban/rural) and region (South/Center/North). Comparisons between 2005 and 2014/2015 data were made, for 10-year age-group estimates, and for the 25-64 age-group (after direct standardization, using the 2005 population as reference). Supplementary Material (Table S1; https://cadernos.ensp.fiocruz.br/static//arquivo/suppl-e00029024_5232.pdf) shows the nonstandardized estimates for 2014/2015. The analyses were conducted using Stata, version 15.1 (https://www.stata.com), considering the sampling weights, to ensure that computed prevalence estimates reflect the frequencies in Mozambique, adjusting for stratification by province and clustering at the primary sampling unit level.

### Ethics

The protocols were approved by the Mozambican National Ethics Committee (Ref.: 86/CNBS/2005) and participants provided written informed consent.

## Results

### Prevalence of daily tobacco consumption

Between 2005 and 2014/2015, there was a significant decrease in the prevalence of daily tobacco consumption among older women and men (45-64 years), and among younger women (25-34 years). There was a decrease in daily consumption among those with lower education (from 25% to 12.8% and from 50.8% to 36.6% among women and men, respectively), rural dwellers (from 19.8% to 11.1% and from 40.5% to 31.5% among women and men, respectively) and among urban men (from 27.3% to 19.2%). Although a decrease in the daily consumption of tobacco products in all Mozambican regions was found, the differences were only statistically significant in the North, between women (from 23.5% to 12.2%) and men (from 40.7% to 30.1%), and in the South, among men (from 25.3% to 12.3%) (Table 1).

**Table 1:** Prevalence * of daily tobacco consumption in Mozambique (2005 and 2014/2015).

Variables	Women [% (95%CI)]			Men [% (95%CI)]		
	2005	2014/2015	p-value	2005	2014/2015	p-value
Age (years)						
25-34	7.5 (4.3-12.7)	2.0 (1.0-4.0)	0.016	32.7 (28.0-37.7)	26.8 (20.9-33.7)	0.150
35-44	14.6 (10.1-20.8)	15.1 (9.9-22.2)	0.904	35.3 (29.2-41.9)	34.1 (25.4-44.0)	0.835
45-54	27.6 (20.8-35.7)	10.3 (7.0-14.9)	0.000	36.6 (29.0-45.0)	23.0 (16.2-31.6)	0.013
55-64	34.0 (24.9-44.5)	17.4 (11.2-26.1)	0.008	45.3 (31.2-60.3)	23.7 (15.7-34.1)	0.014
Education (years)						
< 1	25.0 (19.9-30.8)	12.8 (9.1-16.5)	0.000	50.8 (41.4-60.1)	36.6 (27.3-45.9)	0.035
1-5	10.1 (7.6-13.3)	6.9 (3.9-9.9)	0.130	37.1 (32.0-42.5)	30.5 (24.2-36.8)	0.115
≥ 6	4.1 (1.8-9.0)	3.4 (1.0-5.8)	0.751	20.7 (15.3-27.3)	21.8 (16.5-27.1)	0.788
Place of residence						
Urban	8.0 (5.7-11.1)	5.1 (3.1-7.0)	0.088	27.3 (22.7-32.4)	19.2 (13.7-24.6)	0.030
Rural	19.8 (15.3-25.1)	11.1 (8.2-13.9)	0.003	40.5 (33.7-47.6)	31.5 (26.2-36.8)	0.044
Region						
South	5.1 (3.4-7.7)	2.3 (0.5-4.1)	0.050	25.3 (19.3-32.3)	12.3 (6.7-17.9)	0.003
Center	6.5 (4.4-9.4)	4.8 (2.5-7.0)	0.322	31.5 (27.4-35.9)	27.8 (22.4-33.3)	0.294
North	23.5 (18.2-29.6)	12.2 (9.1-15.2)	0.001	40.7 (33.1-48.8)	30.1 (24.4-35.8)	0.032

95%CI: 95% confidence interval.

* Data from the 2014/2015 survey were age-standardized (except for prevalence by age strata), using the 2005 population as reference and 10-year age-groups.

### Prevalence of daily tobacco smoking

Over the 10-year period, there was a significant decrease in the prevalence of daily tobacco smoking among women of all age groups, except for those aged 35-44 years, while the decrease among men was limited to those aged 45-54 years. There was also a significant decrease in the prevalence among women with lower education and from rural areas. Among Northern women and Southern men, the prevalence of daily tobacco smoking decreased from 14.9% to 5.1% and from 25% to 12.3%, respectively (Table 2).

**Table 2:** Prevalence * of daily tobacco smoking ** in Mozambique (2005 and 2014/2015).

Variables	Women [% (95%CI)]			Men [% (95%CI)]		
	2005	2014/2015	p-value	2005	2014/2015	p-value
Age (years)						
25-34	5.7 (2.6-11.8)	0.9 (0.3-2.1)	0.045	32.5 (27.8-37.6)	26.8 (20.9-33.7)	0.166
35-44	9.2 (4.9-16.6)	5.8 (3.5-9.6)	0.312	34.1 (27.9-40.8)	34.1 (25.4-44.0)	1,000
45-54	10.8 (6.5-17.3)	3.9 (1.9-7.9)	0.029	33.4 (26.8-40.6)	23.0 (16.2-31.6)	0.049
55-64	19.1 (10.4-33.5)	6.5 (3.2-12.8)	0.048	35.7 (24.9-48.1)	21.9 (13.7-33.2)	0.074
Education (years)						
< 1	14.3 (8.9-22.1)	4.5 (2.5-6.5)	0.005	46.4 (37.8-55.2)	36.6 (27.3-45.9)	0.131
1-5	5.4 (3.4-8.5)	3.5 (1.6-5.4)	0.242	34.6 (30.0-39.4)	30.0 (23.6-36.4)	0.256
≥ 6	2.8 (1.0-7.4)	0.7 (0.0-1.6)	0.212	19.9 (14.6-26.6)	21.8 (16.5-27.1)	0.642
Place of residence						
Urban	3.2 (2.0-5.2)	3.1 (1.4-4.7)	0.932	27.1 (22.6-32.1)	19.2 (9.4-29.0)	0.155
Rural	11.7 (7.3-18.1)	3.7 (2.2-5.3)	0.005	36.8 (30.7-43.3)	31.0 (25.6-36.4)	0.171
Region						
South	2.0 (0.1-5.0)	1.4 (0.0-2.7)	0.674	25.0 (18.8-32.3)	12.3 (6.7-17.9)	0.005
Center	0.7 (0.2-2.1)	1.0 (0.2-1.9)	0.645	30.1 (25.6-35.0)	27.8 (22.4-33.3)	0.531
North	14.9 (9.9-21.7)	5.1 (3.2-7.0)	0.002	37.2 (30.5-44.3)	29.7 (23.9-35.5)	0.103

95%CI: 95% confidence interval.

* Data from the 2014/2015 survey were age-standardized (except for prevalence by age strata), using the 2005 population as reference and 10-year age-groups;

** The proportion of daily tobacco smokers in each category may not be equal to the sum of the proportions of manufactured and hand-rolled cigarettes due to rounding or because the former also included the daily consumption of other tobacco products (pipe, shisha and cigar). Moreover, in the 2014/2015 survey, daily smokers can be daily users of both manufactured and hand-rolled cigarettes.

**Figure 1:**
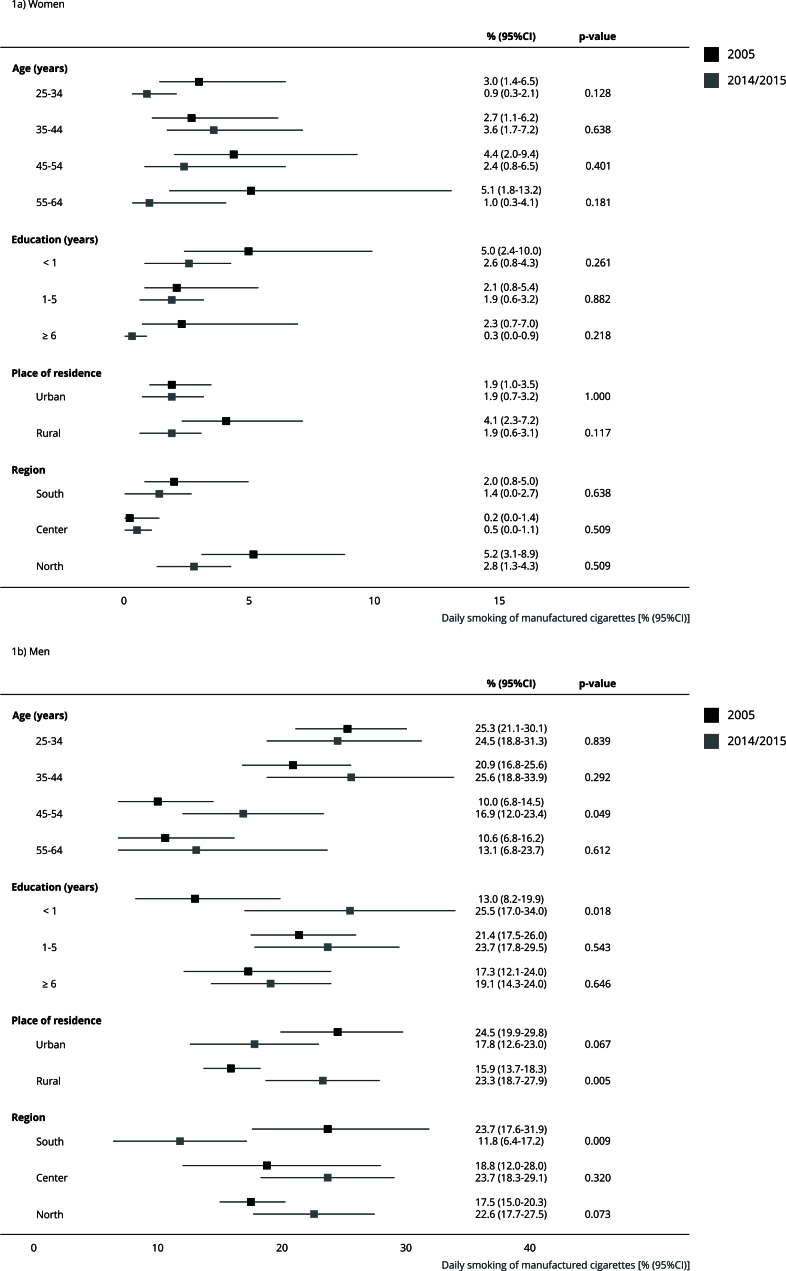
Prevalence * of daily smoking of manufactured cigarettes in Mozambique (2005 and 2014/2015).

Concerning the consumption of manufactured cigarettes, there was a significant increase among those men aged 45-64 years (from 10% to 16.9%), illiterate (from 13% to 25.5%) and from rural areas (from 15.9% to 23.3%); whereas it decreased among Southern men (from 23.7% to 11.8%) (Figure 1).

* Data from the 2014/2015 survey were age-standardized (except for prevalence by age strata), using the 2005 population as reference and 10-year age-groups.

**Figure 2:**
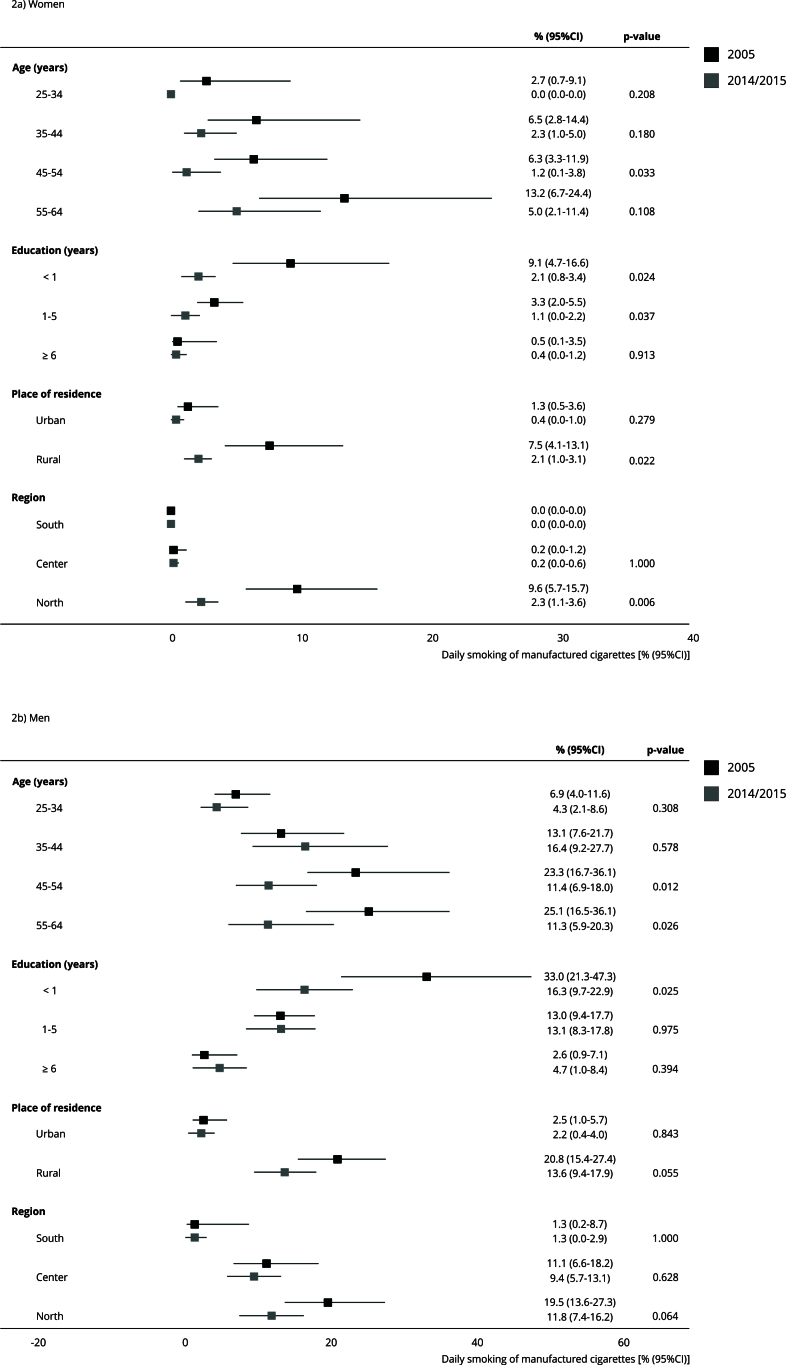
Prevalence * of daily smoking of hand-rolled cigarettes in Mozambique (2005 and 2014/2015).

Regarding the daily consumption of hand-rolled cigarettes, a decrease was found among women aged 45-54 years (from 6.3% to 1.2%), with lower education (from 9.1% to 2.1% in those with < 1 year of education and from 3.3% to 1.1% in those with 1-5 years of education), from rural areas (from 7.5% to 2.1%) and from the North (from 9.6% to 2.3%). Among men, consumption decreased only for those aged 45-64 years (from 23.3% to 11.4% in those aged 45-54 years and from 25.1% to 11.3% in those aged 55-64 years) and in those with lower education (from 33% to 16.3%) (Figure 2).

* Data from the 2014/2015 survey were age-standardized (except for prevalence by age strata), using the 2005 population as reference and 10-year age-groups.

### Prevalence of daily use of smokeless tobacco

The daily consumption of smokeless tobacco decreased among women aged 45-54 years (from 17.1% to 6.7%) and among women from the South (from 3.1% to 1%). Among men, there was a decrease in the oldest age group (from 13.4% to 1.8%), in the less educated (from 7.7% to 0.3%) and in those from rural areas (from 5.1% to 1.5%). In 2014/2015, there were no daily users of smokeless tobacco among men from urban areas and from the South and Center regions (Table 3).

**Table 3:** Prevalence * of daily use of smokeless tobacco in Mozambique (2005 and 2014/2015).

Variables	Women [% (95%CI)]			Men [% (95%CI)]		
	2005	2014/2015	p-value	2005	2014/2015	p-value
Age (years)						
25-34	2.1 (1.0-4.5)	1.1 (0.4-3.2)	0.382	0.2 (0.0-1.2)	0.3 (0.0-2.0)	0.867
35-44	5.5 (3.3-9.0)	10.6 (5.7-18.8)	0.162	1.6 (0.6-4.2)	1.4 (0.3-6.5)	0.913
45-54	17.1 (11.5-24.6)	6.7 (4.1-10.7)	0.005	4.5 (1.9-10.2)	1.2 (0.2-8.6)	0.273
55-64	16.5 (10.3-25.3)	12.2 (6.9-20.7)	0.408	13.4 (7.4-23.0)	1.8 (0.3-11.3)	0.017
Education (years)						
< 1	11.2 (6.8-18.0)	9.1 (5.2-12.9)	0.545	7.7 (3.5-16.0)	0.3 (0.0-1.0)	0.021
1-5	4.9 (3.1-7.7)	4.0 (1.4-6.5)	0.607	3.0 (1.5-5.8)	1.8 (0.0-3.8)	0.412
≥ 6	1.8 (5.4-5.7)	2.7 (0.5-4.9)	0.424	0.7 (0.2-3.2)	0.2 (0.0-0.5)	0.519
Place of residence						
Urban	4.9 (3.2-7.3)	2.7 (1.1-4.3)	0.097	0.2 (0.0-1.4)	-	-
Rural	8.5 (5.1-13.9)	7.8 (4.9-10.8)	0.796	5.1 (3.0-8.3)	1.5 (0.1-2.8)	0.018
Region						
South	3.1 (1.9-5.2)	1.0 (0.0-2.1)	0.035	0.3 (0.0-2.7)	-	-
Center	6.3 (4.3-9.2)	4.3 (2.1-6.5)	0.234	1.9 (0.8-4.5)	-	-
North	9.0 (5.0-15.6)	7.6 (4.6-10.7)	0.654	4.9 (2.6-8.8)	1.5 (0.1-2.9)	0.050

* Data from the 2014/2015 survey were age-standardized (except for prevalence by age strata), using the 2005 population as reference and 10-year age-groups.

## Discussion

Over the 10-year period, a significant reduction was observed in the prevalence of daily consumption of hand-rolled cigarettes among Northern women, and of manufactured cigarettes among Southern men. Conversely, although not statistically significant, there was an increase in the consumption of manufactured cigarettes among men from the Center and North regions. Regarding smokeless tobacco, there was a decrease in consumption among women in the Southern region. In 2014/2015, there were no daily users among men from urban areas and from the South and Center regions.

In 2005 and 2014/2015, traditional forms of tobacco use, specifically hand-rolled cigarettes and smokeless tobacco, were more prevalent in the Northern provinces of Mozambique. This could be partially explained by proximity to tobacco production areas (most of the tobacco leaf production in Mozambique is concentrated in the Northern portion ^8^) and the fact that northern dwellers are generally poorer and less educated than their counterparts ^7^. In fact, previous studies reported that lower education is associated with higher prevalence of smoking and smoking cessation is also lower among less educated citizens compared to those who are highly educated ^14^ ^,^ ^15^. While there are slight differences in how provinces were grouped (North or South in the previous study versus South, Center, or North in ours) and in the outcomes evaluated (current consumption in the previous study versus daily consumption in ours), the regional differences found are in line with findings from a national household survey conducted in 2003 that evaluated a representative sample of Mozambicans aged 25-64 years ^16^. Regarding consumption of hand-rolled cigarettes, our study found that daily consumption decreased in both sexes in the North between 2005 and 2014/2015, with statistically significant differences observed among women. The shift to westernized forms of tobacco consumption may, at least partly, explain the decrease among males, since the prevalence of manufactured cigarettes increased in the Northern provinces. However, it does not explain the decrease observed in women, as daily consumption of manufactured cigarettes also decreased in this group. From 2008 to 2014, there was a reduction in poverty in almost all provinces except for the provinces of Niassa, Nampula and Cabo Delgado, where the proportion of citizens in poverty increased from 33% to 60.6%, from 51.4% to 57.1%, and from 39% to 44.8%, respectively ^17^. Such impoverishment may be related with the floods that affected these provinces in the second trimester of 2014, leading to significant crop loss, which may have contributed to the reduction in the consumption of hand-rolled cigarettes among women. In fact, although the northern societal structure is predominantly matrilineal, previous evidence has shown that this does not automatically guarantee gender equality, and many women still lack access to income and remain financially dependent on men ^18^. It is important to continue monitoring the potential impact of the economic development of this region in tobacco consumption patterns for a timely implementation of measures to control tobacco consumption.

In Southern provinces, which represent the most westernized part of the country, there was a significant halving of the daily consumption of manufactured cigarettes among men. Conversely, in the Center and North regions, a nonsignificant increase was observed (from 18.8% to 23.7% and from 17.5% to 22.6%, respectively). In addition, in contrast to results from a previous study conducted in Mozambique in 2003 - reporting a prevalence of current smoking of manufactured cigarettes among men approximately 30% higher in Maputo City, Maputo, Inhambane and Gaza provinces when compared to Northern provinces ^16^ - our study found a nearly twice higher daily consumption of manufactured cigarettes in 2014/2015 in Northern and Center provinces when compared to the Southern. This shift among men may indicate a movement towards the consumption of more westernized forms of tobacco in traditionally less westernized provinces. On the other hand, it might also suggest a transition to a new phase of tobacco epidemic in the more developed regions of the country. Between 2005 and 2014/2015, the observed decrease in daily consumption of manufactured cigarettes in the South could be attributed to the increase in tobacco prices in 2013-2016, reducing affordability ^19^, or to the positive effects of the Regulation of Consumption and Marketing of Tobacco approved by the government in 2007 ^20^. This seems to have had a higher impact in the South when compared to Center and Northern regions, where a nonsignificant increase was observed among men. The contribution of the illicit cigarette trade in Mozambique ranged between 1% and 2% of the total consumption in 2012, while in other countries of Southern Africa it was estimated at 10%-23% ^21^. As the neighboring countries have much higher cigarette prices and taxes, cigarette smuggling into Mozambique is very unlikely. The disparity in daily manufactured cigarette consumption patterns across various regions highlights the need for targeted tobacco control strategies tailored to each region. Especially for the central and northern provinces, in which cigarette consumption has a tendency to increase. Implementing public health campaigns and strengthening regulatory measures can be crucial to mitigate the potential rise in cigarette consumption.

The WHO Framework Convention on Tobacco Control (FCTC), an international legal agreement with 182 parties, encompasses measures such as taxation, smoke-free environments, advertising bans, graphic warning labels, and age restrictions ^22^. Mozambique only ratified the FCTC in 2017 ^23^, therefore, it seems unlikely that it had a direct impact on our results. However, there are challenges in implementing the FCTC, like the relevant contribution of tobacco to the total export value of agricultural commodities and the increase of the tobacco industry in corporate social activities ^8^ ^,^ ^24^. The latter may enhance the industry’s reputation and contribute to weaken and delay tobacco control efforts in Mozambique, similarly to what has been described in other sub-Saharan countries ^24^ ^,^ ^25^ ^,^ ^26^. According to the last FCTC implementation progress report, Mozambique reported measures to achieve all FCTC articles, except for article 5.3, which included measures to prevent tobacco industry interference in public health policies ^23^. Over the past two decades, Mozambique has substantially increased its tobacco leaf production, becoming the third highest exporter among African countries in 2018 ^27^. Future studies are needed to extend the analysis of tobacco consumption in Mozambique beyond 2015 and to understand the impact of the WHO FCTC ratification on the consumption of tobacco products in Mozambique.

A 4-stage model of the tobacco epidemic in developed countries was proposed in 1994 by Lopez et al. ^28^ as an attempt to predict tobacco consumption but also health-related disorders. It considered the prevalence of smoking (regular smoking in the adult population), the amount of cigarettes smoked per adult and the mortality due to smoking. However, this model cannot be directly applied to developing countries ^29^. For instance, in Mozambique - as in other sub-Saharan countries - traditional forms of tobacco were used for a long time before the arrival of western tobacco products. Also, a higher use of tobacco by groups with low education in the early stages of the tobacco epidemic, mostly due to the predominance of traditional forms of tobacco consumption, contrasts with what happened in most developed countries, in which tobacco consumption occurred among the more educated population first.

This study, based on the evaluation of large representative samples of the Mozambican adults, provides the best available evidence regarding the trends of daily use of smoked and smokeless tobacco, across different Mozambican regions. However, some limitations need to be discussed. Firstly, methodological differences between the 2005 and the 2014/2015 STEPS surveys need to be considered. Particularly, the 2005 STEPS survey classified daily users of more than one type of smoked or smokeless tobacco, as applicable, as consumers of the type they used most frequently. This could have led to an underestimation of the prevalence of daily consumers of manufactured and hand-rolled cigarettes in 2005, as well as of the differences found between 2005 and 2014/2015, resulting in conservative estimates of the decrease observed in smoking prevalence in the 10-year period. Conversely, it may have caused an overestimation of the increase in smoking prevalence reported between the two periods (for instance, the increase in the prevalence of manufactured cigarettes in Northern men). This limitation also precluded the comparison of daily consumption of manufactured and hand-rolled cigarettes between the two periods. Furthermore, the lack of data in the 2005 survey for those younger than 25 years prevented the study of the trends in young adults, a vulnerable population for risk factors for noncommunicable diseases in this setting ^30^. Although selling tobacco to those aged under 18 is illegal in Mozambique, we previously demonstrated that in 2014/2015, the proportion of daily smokers who reported having started smoking daily before the age of 25 was approximately half the number of daily smokers in urban areas, while in rural settings, it was more than one-third of the number of women and more than half of men ^6^. Therefore, future studies evaluating tobacco consumption in Mozambique should include this age strata in order to provide a better understanding of the tobacco-related burden expected for the next years in the country.

## Conclusion

In conclusion, our study extends our previous reports by revealing a decrease in the daily consumption of hand-rolled cigarettes among women in the Northern region and a decline in the consumption of manufactured cigarettes among men in the Southern region over the same period. Moreover, our findings indicate a potential future trend towards residual smokeless tobacco consumption. The results also suggest increases in the daily consumption of manufactured cigarettes among men in the Center and North regions, which raises concerns regarding future trends. To effectively address the tobacco burden in this setting, future efforts should be integrated into a comprehensive and sustainable strategy, which should account for variations in tobacco consumption within regions and consider the socioeconomic dimensions of tobacco control.
